# Supplementary figures and images for: Metabolic modeling reveals determinants of prebiotic and probiotic treatment efficacy across multiple human intervention trials
Source: PLoS Biol. 2026 Feb 19;24(2):e3003638. doi: 10.1371/journal.pbio.3003638 (PMC12919772; doi:10.1371/journal.pbio.3003638)

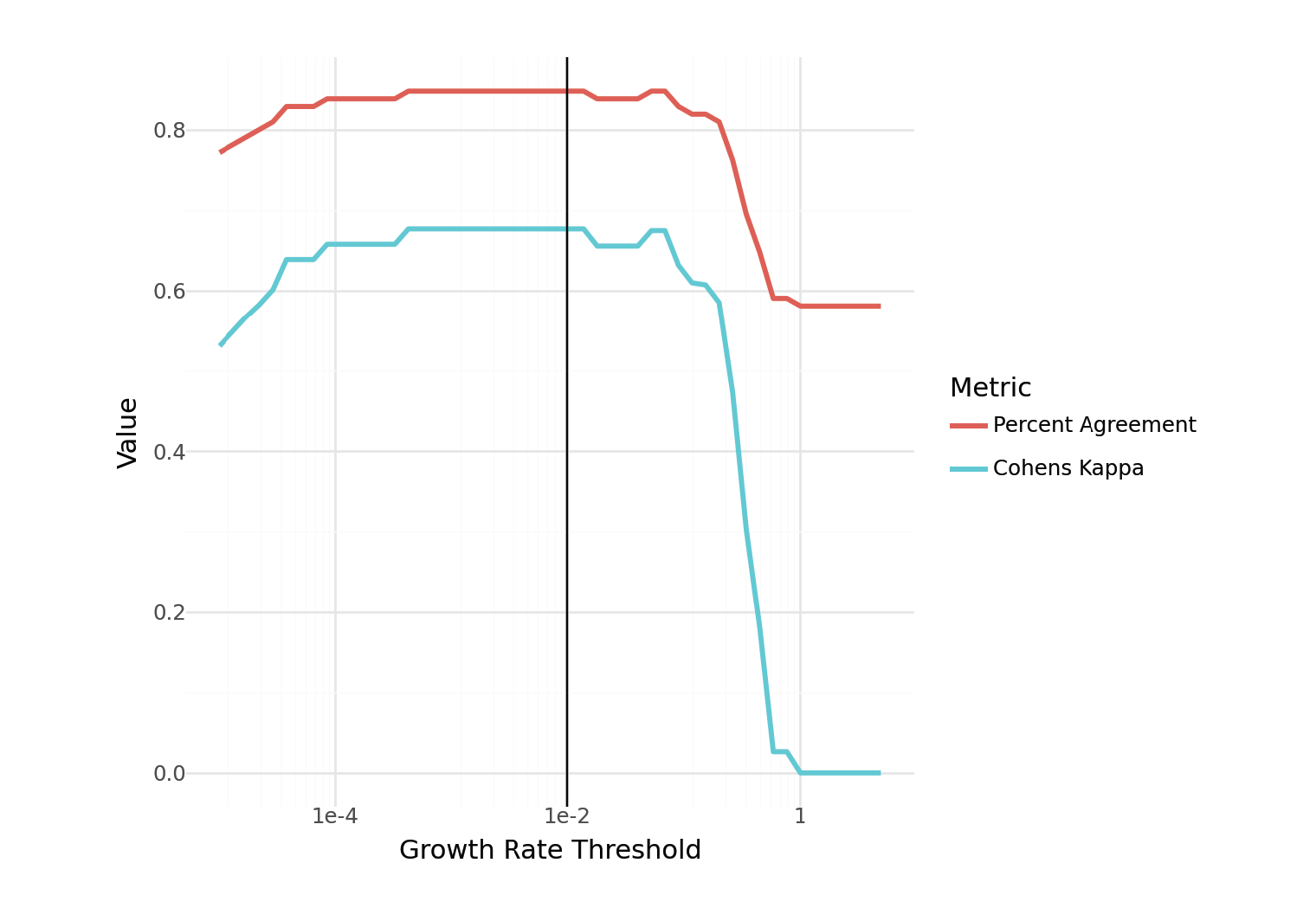

Supplement: S1 Fig — Agreement fraction (red line) and Cohen’s κ (blue line) are shown as a function of the growth rate threshold, around which model predictions are binarized for growth or non-growth. A black vertical line is shown at 0.01, the value used in this analysis, equivalent to doubling time of ~70 hours. Underlying available in S2 Data. (TIFF) [file pbio.3003638.s001.tiff]

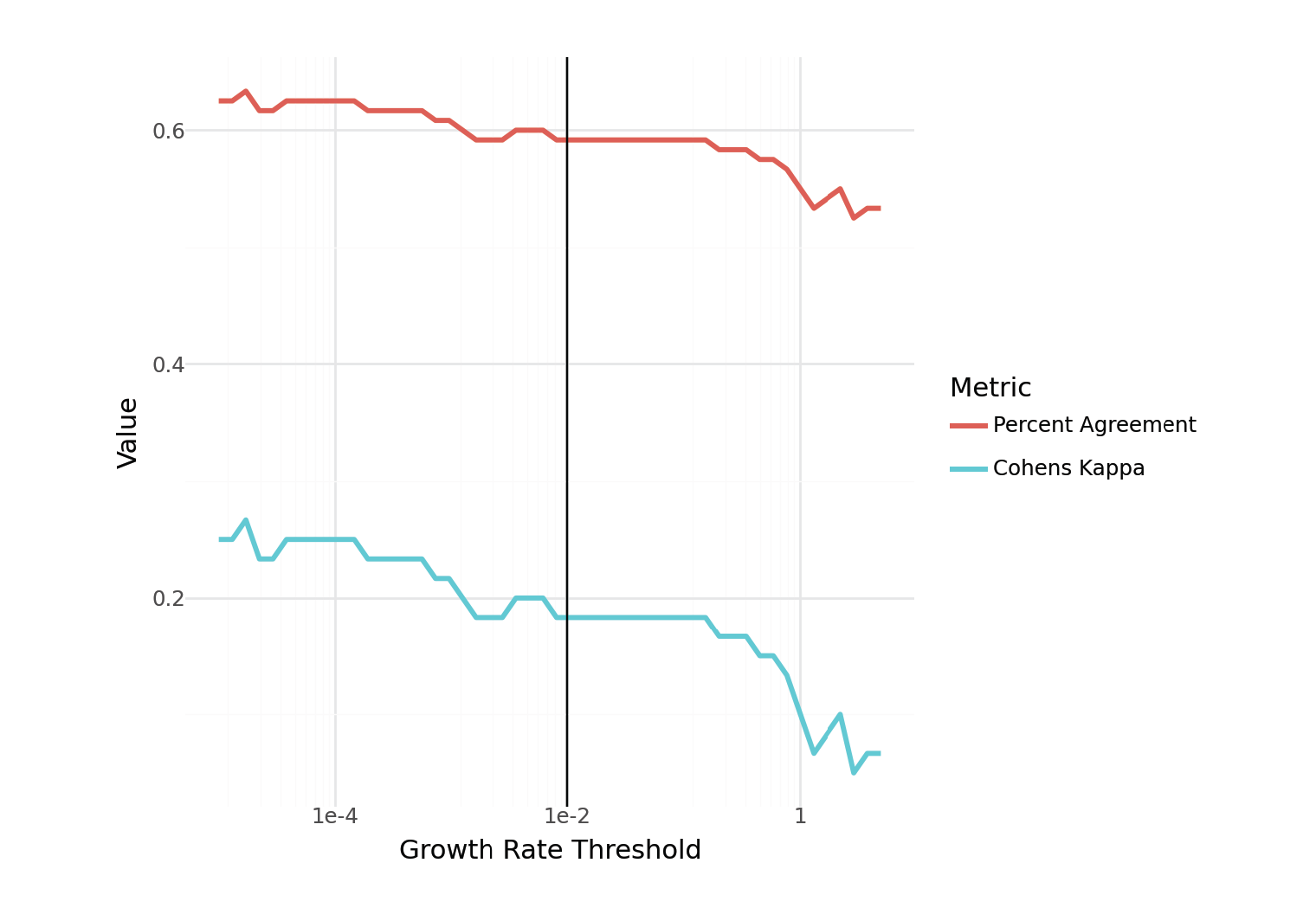

Supplement: S2 Fig — Agreement fraction (red line) and Cohen’s κ (blue line) are shown as a function of the growth rate threshold, around which model predictions are binarized for growth or non-growth. A black vertical line is shown at 0.01, the value used in this analysis, equivalent to doubling time of ~70 hours. Underlying available in S2 Data. (TIFF) [file pbio.3003638.s002.tiff]

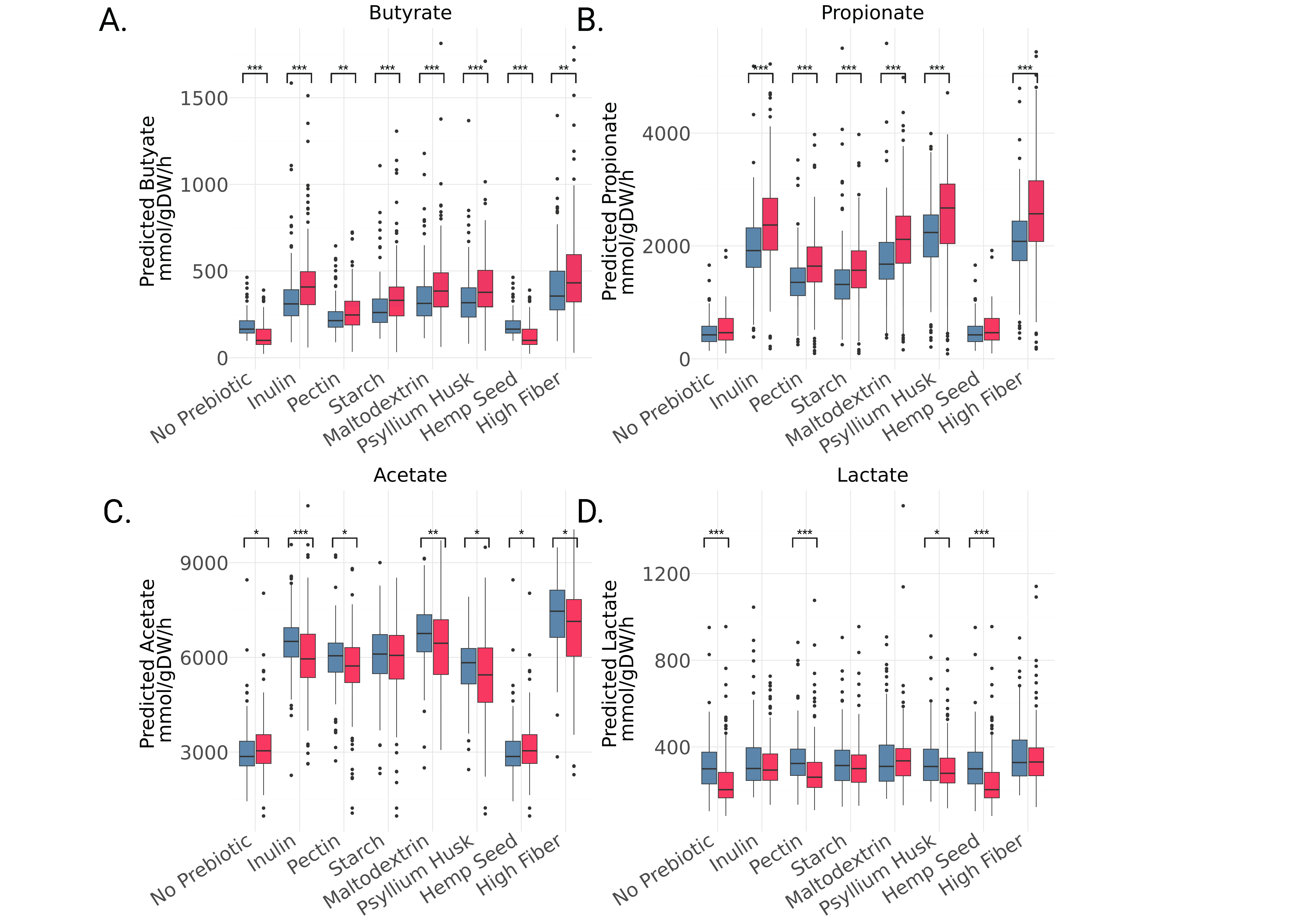

Supplement: S3 Fig — To ensure that observed shifts in metabolite production were not driven by overall changes in biomass, production rates were normalized by predicted community growth rates, which reflect the total biomass produced by the community. Scaled values for SCFA and lactate production were largely consistent with the unscaled predictions across prebiotic and probiotic conditions. Significant differences between ±probiotic treatments within each prebiotic or diet intervention were assessed using a Mann–Whitney U test (p < 0.05; p < 0.01; p < 0.001). Underlying available in S5 Data. (TIFF) [file pbio.3003638.s003.tiff]

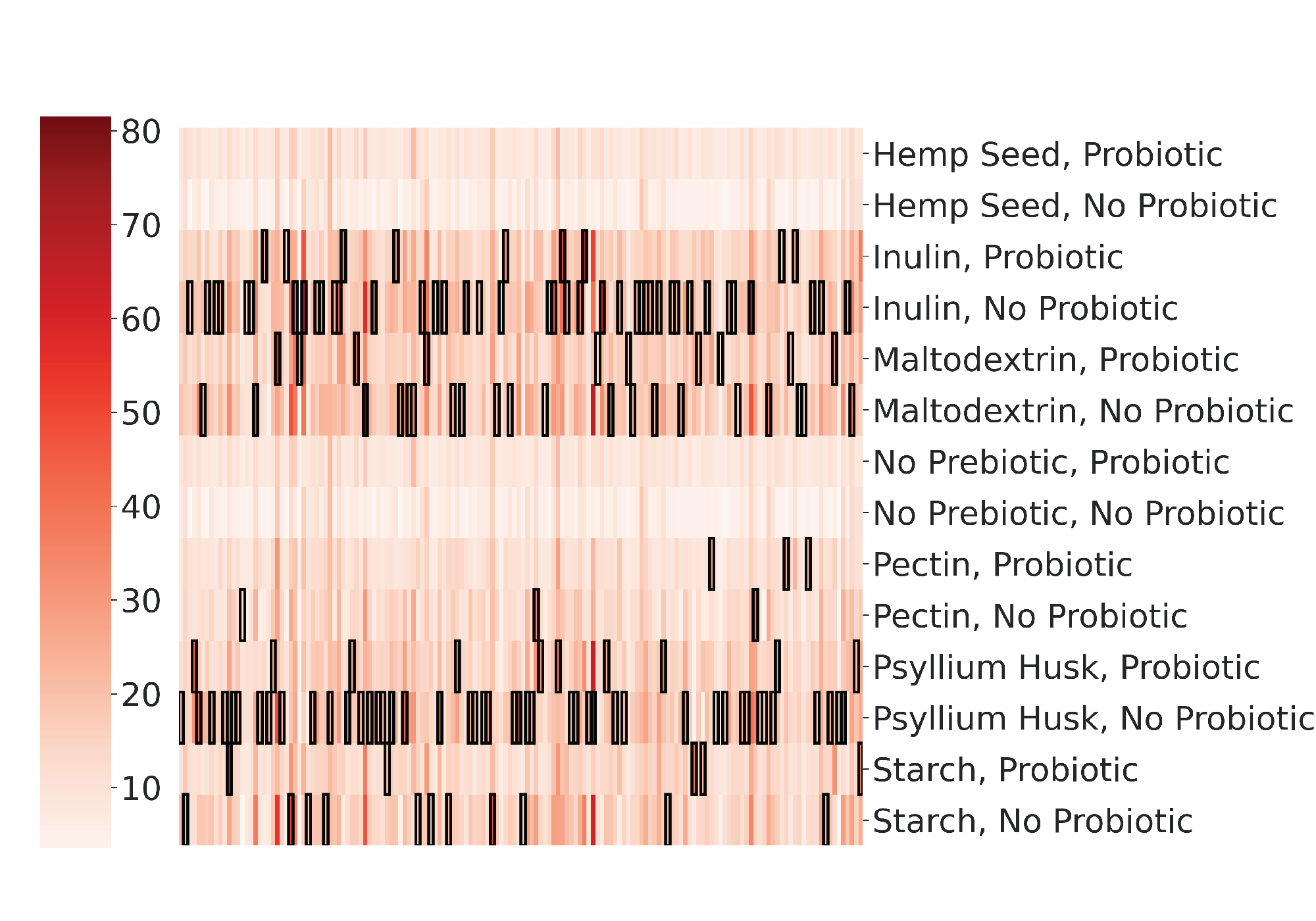

Supplement: S4 Fig — MCMM-predicted butyrate production varied substantially across individuals (N = 156) in response to different prebiotic and probiotic combinations. The combination of psyllium husk with no probiotic cocktail produced the highest butyrate flux in the greatest number of individuals. However, every prebiotic/probiotic combination was optimal for at least one individual, with the exceptions of: (1) hemp seed, and (2) the no-prebiotic condition. Heatmap colors indicate predicted butyrate flux (mmol/gDW/h), and black boxes denote the most effective treatment for each sample. Underlying available in S5 Data. (TIFF) [file pbio.3003638.s004.tiff]

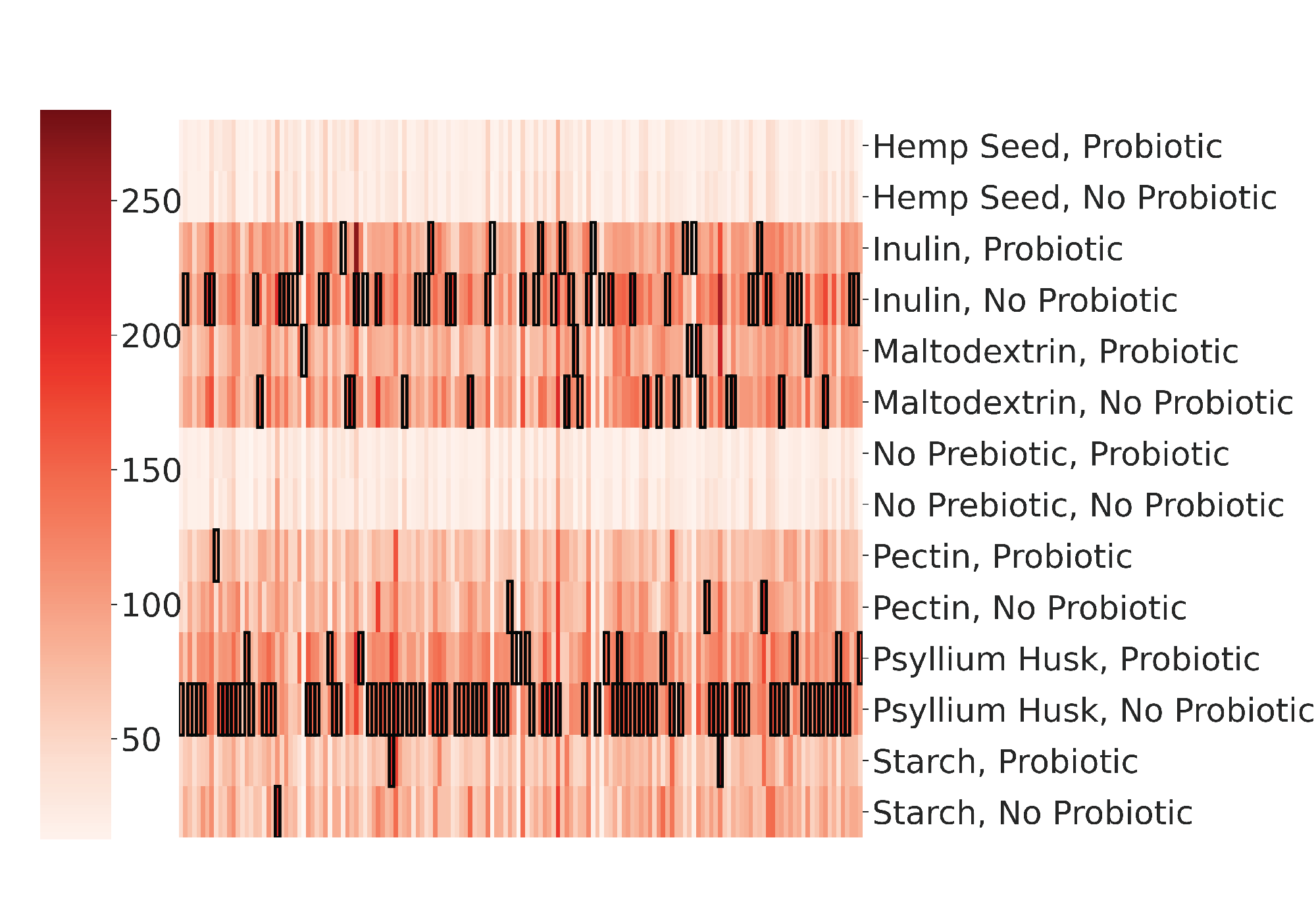

Supplement: S5 Fig — MCMM-predicted propionate production also varied substantially across individuals (N = 156) in response to different prebiotic and probiotic combinations, though slightly less than for butyrate. The combination of psyllium husk without a probiotic cocktail produced the highest propionate flux in the greatest number of individuals. However, every other prebiotic/probiotic combination was optimal for at least one individual, with the exceptions of: (1) hemp seed, and (2) the no-prebiotic condition. Heatmap colors indicate predicted propionate flux (mmol/gDW/h), and black boxes denote the most effective treatment for each sample. Underlying available in S5 Data. (TIFF) [file pbio.3003638.s005.tiff]
