# Supplementary material for: Metabolic modeling reveals determinants of prebiotic and probiotic treatment efficacy across multiple human intervention trials
Source: PLoS Biol. 2026 Feb 19;24(2):e3003638. doi: 10.1371/journal.pbio.3003638 (PMC12919772; doi:10.1371/journal.pbio.3003638)
Supplement: Attachment — Submitted filename: reviewer_response.docx [file pbio.3003638.s013.docx]

Summary: We thank the reviewers for their thoughtful comments. We have made major revisions to our manuscript to address each reviewer concern, and you can find our point-by-point responses below. In particular, we have included a second human intervention trial to further validate MCMM engraftment predictions in a totally independent context. We have also included a longitudinal analysis of the Arivale cohort, indicating that changes in MCMM-predicted butyrate, when switching from a low- to a high-fiber model diet, are significantly associated with beneficial changes in clinical chemistries following a real-world high-fiber dietary intervention. We believe these revisions have substantially increased the robustness and impact of our work.

REVIEWS:

Reviewer's Responses to Questions

Reviewer #1: This study explores how microbial community-scale metabolic models (MCMMs) can predict the success of synbiotic interventions (combinations of probiotics and prebiotics) in human subjects. The authors used data from a placebo-controlled trial where participants received a synbiotic cocktail (WBF-011) containing five probiotic strains and inulin, a prebiotic fiber. The authors highlight the potential of MCMMs as tools for precision microbiome therapeutics, capable of customizing interventions based on an individual's microbiome composition and dietary background. This manuscript presents an innovative and timely contribution to microbiome research and precision health. With more rigorous discussion of modeling limitations, clarification of the clinical relevance of findings, and restrained interpretation of personalization claims, this work will be a valuable addition to the literature.

Comments:

1. While the authors report >85% accuracy for predicting probiotic engraftment using MCMMs, the extent of external validation is limited. Given the complexity and heterogeneity of gut microbiota and host factors, it is critical to test the model's robustness across independent cohorts or datasets. The use of a secondary observational cohort is a good start but lacks the depth of validation necessary for broader translational claims. Please include additional validation analyses, or clearly state the exploratory nature of the personalization findings and their limitations.

Response: We acknowledge that the validation presented in the initial manuscript (Validation Study A), while encouraging, was limited to a single cohort. In recognition of the need for more rigorous testing of model generalizability, we have incorporated a second independent cohort (Validation Study B), comprising 20 participants treated with an 8-species probiotic cocktail, called VE303 (Figs. 1-2). While this additional analysis provides an independent test of the model, we recognize that the cohort size and specific intervention may not fully capture the diversity of host–microbiome interactions observed across broader populations. As such, we frame our personalization findings as exploratory and emphasize in the revised text the importance of future work involving larger, more heterogeneous cohorts, varied probiotic formulations, and prospective multi-site studies to robustly assess predictive accuracy and translational applicability (Lines 366-370).

2. MCMMs rely on several simplifying assumptions (e.g., steady-state flux balance, known pathways, representative genomes) that limit their ability to capture host-specific microbiome dynamics. The discussion section does not adequately address these model constraints. Include a more nuanced discussion of model limitations and assumptions, and their potential impact on prediction accuracy and biological interpretation.

Response: The reviewer brings up an important point about the implicit assumptions that come along with any analysis involving metabolic modeling. A more thorough discussion revolving around these assumptions, as well as their potential implications with regard to limitations of the model, has been added to the revised manuscript (Lines 338-348)

3. The role of diet in modulating SCFA production and probiotic colonization is critical, yet the manuscript provides little information on participants' dietary intake or control during the intervention. Please clarify whether dietary data were collected and how variation in intake was accounted for in the models and analysis.

Response: We agree with the reviewer that diet is of primary importance with regard to the growth behavior of the microbiome and its metabolic production. Unfortunately, adequate dietary intake data for the cohorts included in this study were missing. We have added an in depth discussion of this point in the discussion section, detailing the approach taken to overcome the missing data (i.e., assuming everyone was consuming a standard European diet, as defined on the Virtual Metabolic Human database), and the potential pitfalls of this approach (349-352).

4. The use of MCMMs to propose personalized synbiotic formulations is promising but currently remains theoretical. There is a risk of overstating real-world readiness. We should acknowledge this as a proof-of-concept and clearly delineate what further steps would be required to operationalize personalization clinically.

Response: We thank the reviewer for this critique, as it prompted us to devise a longitudinal analysis to further derisk the translational potential of our approach. We agree that the current work should be framed as a proof-of-concept rather than a clinically-ready application. We have added an acknowledgement of this in the discussion (lines 362-366). The revised text now clarifies that while MCMMs show promise for rational and personalized synbiotic design, further validation in larger, heterogeneous cohorts and prospective precision trials will be essential to establish clinical readiness (lines 366-370). However, as mentioned above, we now include a longitudinal analysis of the Arivale cohort, which was given a healthy diet and lifestyle intervention (i.e., individuals were coached to increase their dietary fiber intake and their clinical chemistries were tracked over time). We built MCMMs for individuals with longitudinal response data, where we predicted personalized changes in butyrate production when going from a low- to a high-fiber diet within the model. We then regressed these predicted changes in butyrate against changes in 127 clinical chemistries, controlling for age, sex, and baseline values for each respective chemistry. 9 chemistries showed significant associations with changes in MCMM-predicted butyrate production (FDR q < 0.05), including improvements in insulin sensitivity and reduced immune activation with greater increases in butyrate production (Fig. 4; lines 197-212 & 317-327). This retrospective analysis provides strong evidence for the clinical applicability of our predictions, but we also recognize that further validation in prospective trials is needed to solidify the translational relevance of MCMM–optimized interventions.

5. Please pay attention to spelling and grammar issues. For example, in line 75, "date" should be spelled "data".

Response: Thank you for calling this to our attention - we have corrected this typo, and we have completed a thorough proofread for other spelling and grammatical errors.

6. The authors use qPCR but the results are not quantitative outside of using the inverse of the cycle threshold. While this is used as a proxy, the value of this approach is questionable. As the data is presented, the authors are using PCR which is semiquantitative.

Response: We thank the reviewer for highlighting this important point. Unfortunately, the standards used for absolute quantification were not retrievable upon reaching out to the study authors, so the qPCR data are now presented as semiquantitative. To improve the interpretability of the data, we now present the data as -Ct, but without the standards we are indeed limited to a semi-quantitative interpretation of the data. We have clarified this limitation in the revised manuscript and emphasize that while absolute abundances cannot be determined, these relative measures remain informative for assessing trends in microbial growth and evaluating model predictions within the scope of this proof-of-concept study (423-428).

7. When performing qPCR, a standard curve should be run so the results will provide a quantitative amount. Furthermore, when using something like SYBR green, the melt curve of samples should match the standard to give confidence in target detection. This would provide additional data as it relates to the comment in lines 101-103. While the authors provide a threshold for some low abundance bacteria, I don't think there is any information on how this was generated or validation data provided.

Response: Unfortunately, the standard curves used for absolute quantification were not available, and the resulting qPCR data are therefore presented as semiquantitative, using -Ct as a proxy for relative abundance. We agree that standard curves and melt curve validation data would have been ideal here. While melt curve data were not available from the original authors of the article, all primers used were validated for target specificity (Perraudeau et al., 2020). We have clarified these limitations in the revision and emphasized that the qPCR results are used here to capture relative trends in growth rather than absolute quantification (lines 423-428).

8. What is the difference between detection threshold and engraftment threshold? (lines 96-7 vs 112)

Response: In this case, ‘detection threshold’ was used incorrectly, and should instead have been referred to as ‘engraftment threshold.’ This mistake has been corrected in the revised manuscript.

9. You provide predicted values for butyrate and propionate production from models for groups 1-4, but not group 5 (where only statistics are provided) - please provide the data.

Response: We thank the reviewer for pointing out this oversight - it has been fixed to describe the predicted butyrate and propionate values for each group (lines 139-149)

10. Furthermore, detection of the species does not equate to engraftment. Detection over time does suggest that there is colonization.

Response: We appreciate the reviewer bringing up this point, as it is indeed central to the analysis. While we agree that the ideal outcome would be detection over the long-term, sufficient follow-up data for this cohort is not publicly available. As such, the best metric available to analyze for probiotic engraftment is necessarily short-term enrichment. While it is true that short-term enrichment does not equate to engraftment, relative enrichment of certain species (e.g., *B. longum, A. muciniphila*), relative to baseline, likely indicates some degree of *in situ* growth, given the high amount of residual biomass in stool (10^11^-10^12^ bacterial cells per gram of feces), which is unlikely to be impacted directly by the relatively low bacterial biomass present in the probiotic capsules. While perhaps not direct evidence for stable engraftment, it does point toward short-term enrichment of these species. A more thorough discussion of this has been added to the manuscript (lines 267-278).

11. The github repository for codes used in this work seems to be inaccessible.

Response: We apologize for this oversight - the Github repository has been made

publicly available.

------------------

Reviewer #2, Christoph Kaleta (note, reviewer 2 has signed this review): The study by Quinn-Bohnmann & Gibbons shows how to use metabolic modeling to predict engraftment efficiency of probiotics by the supplementation of prebiotics. They are able to predict 85% of engraftments outcomes in a synbiotic study of diabetes patients - an impressive accuracy, given the limited amount of information integrated in the modeling approach. Further, they use the metabolic modeling framework to predict SCFA production in the microbial communities and how this correlates with the systemic inflammation marker CRP in the cohort. Finally, they explore engraftment predictions in another larger cohort using different prebiotic treatments and explore how a personalized synbiotic treatment could look like, if the goal is to increase SCFA production.

I think this is an elegant study using metabolic modeling to predict synbiotic efficiency in a simple cohort design. It shows the power of metabolic modeling in clinical contexts. However, I feel that the second exploratory part with the second cohort is a little detached from the initial simulations and, though interesting as a case study, with little experimental support and could be shortened and better discussed. Other than that, I found some unclear points in methodology and have made some comments to improve the extent of data analysis, to fully understand the results.

Major points:

1. Fig1: Sample size in the plot looks odd, especially at the first time point where often only one or no sample was available. Why is that, can you explain? How were statistics derived for these cases?

Response: We thank the reviewer for pointing this out. The apparent low sample counts at the first time point reflect the fact that the probiotic strain being quantified had not yet been administered and the taxon was not detected (i.e., the full set of samples were included, but many of the Ct values were ‘NA’). In principle, no amplification should occur at this baseline, but in a small number of cases we observed low-level signals, likely due to cross-reactivity of the primers with endogenous strains sharing close sequence similarity. We have clarified these points in the figure legend and methods text (421-422). When only one data point was available from the week zero time point, a 1-sample t-test was used to compare that sample to the 12 week time point.

2. Fig4: Why was the same analysis not applied to AUC values of glucose levels, the other endpoint measurements in the study of Perraudeau et al. (especially, after discussing this relationship in l. 261ff)? Were there other measures which have been tested in Perreadeau et al. which might be of interest in this study?

Response: The reviewer raises a good point - replication of initial findings by Perraudeau et al. was not included in the initial manuscript. The updated manuscript now reflects that there is a significantly larger decrease in Δglucose AUC in the treatment group vs.the placebo (Fig. 3C), concordant with significantly higher growth rates for *A. muciniphila* in the treatment group vs. the placebo (Fig. 3D). We also report a negative association between the model-predicted growth rate of *Akkermansia muciniphila* and ΔglucoseAUC (endpoint vs. baseline) within the treatment group (Fig. 3E). We focused on *A. muciniphila* because this taxon was predicted to engraft well, and it differentiated the WBF-011 probiotic, which showed a significant reduction in AUC, from the WBF-010 probiotic that lacked *A. muciniphila*, which did not show a significant AUC difference from the placebo group. GlucoseAUC and CRP were the primary endpoints measured in the study by Perreadeau et al., and only glucoseAUC (in the WBF-011 group) was reported to show a significant difference from placebo (i.e., there were no differences in CRP between treatment groups). This analysis has been added to the revised manuscript (lines 151-164 & 302-316)

3. Fig5 and Fig6: The results presented are interesting, however I wonder, whether general trends are explained by two factors: first the amount of additional carbon atoms provided to the different communities, secondly the overall growth rates of the communities. For the first factor, it looks to me that independent of what is added to the community, it will increase the production of SCFA. That overestimates the specific effect of SCFA production after addition of the compounds and that the main effect is simply the addition of carbon sources. Hence, I would like to see the effect of the specific metabolite addition compared to glucose equivalents - as this would attribute the SCFA production to the specific metabolite and not to increased carbon sources (In this regard, I think hemp seeds are not metabolized at all by the community, comparing it to no prebiotics - could you check, please?). For the second factor, I would argue that it is interesting to see how much more SCFA are produced per unit biomass produced - as one could argue that this ratio is more relevant than overall production. Generally, increased biomass production will increase fluxes of all other by-products. However, it is not clear what effectively is more relevant in physiological conditions, yet I think the analysis is easily done and gives some more insights in the mechanisms at work.

Response: We thank the reviewer for this suggestion. We deliberately did not compare each prebiotic to an equivalent mass of glucose because that would not reflect physiological conditions within the colon. Adding free glucose to a human gut MCMM will trivially increase SCFA flux, because it bypasses the many enzymatic, transport, and ecological bottlenecks that govern fiber utilization *in vivo*. In contrast, structurally distinct fibers (e.g., inulin, resistant starch, resistant maltodextrin) require specific uptake systems and extracellular degradation pathways that limit which taxa can access the carbon and at what rate. Our MCMM framework explicitly constrains substrate uptake by organism-specific metabolic pathways, so predicted SCFA changes reflect metabolizable carbon uptake and community cross-feeding interactions — not merely the total carbon added. Model diets include glucose, but MICOM strips the diet of substrates that are known to be absorbed in the small intestine (using the human Recon3D model), while adding several host substrates to the diet, like mucins and primary bile acids. Thus, we would argue that a glucose-equivalent control would remove the mechanistic differences we aim to capture and would not be a physiologically-appropriate comparator.

We appreciate the reviewer’s suggestion to examine SCFA production normalized by community growth. As recommended, we repeated the analyses shown in Figure 5A–D in a new supplemental figure (Fig. S3, lines 238-241), normalizing predicted SCFA fluxes by the corresponding modeled community growth rate. This analysis evaluates SCFA yield per unit biomass rather than total production. The trends observed were largely unchanged. These results indicate that our original findings were not driven solely by increases in overall community biomass but reflect genuine shifts in metabolic efficiency and pathway utilization. We thank the reviewer for this helpful suggestion and have incorporated the new analysis into the revised manuscript.

Minor

1. l86-89: I would suggest to shorten to "Microbial abundances for focal species were determined using qPCR (see Method)" to increase clarity of the text.

Response: Thanks, this change has been made (line 101)

2. l106-108: "... metagenomic sequencing data from participants in the WBF-011 treatment arm to assess whether MCMMs could predict the engraftment patterns... " this sentence implies that new metabolic models were reconstructed using the metagenomic data. However, from the methods it reads, that the data was "only" matched to AGORA(2) models. Could you please clarify, what has been done exactly and adjust wording please?

Response: We acknowledge that the wording in the methods section was unclear, and apologize for the oversight. For each participant, a community-scale metabolic model of the microbiome was constructed using AGORA2 GEMs as building blocks, rather than *de novo* construction of models from MAGs or isolate genomes. This has been explicitly clarified in the wording (lines 460-463)

3. l110-122/Fig2: Could you provide a sensitivity analysis of the results for the given threshold of 1e-3 for growth in the simulation, please? This would give insights on how important it is to choose the correct threshold and how prone the analysis is to overfitting.

Response: Sensitivity analysis is now provided in the supplementary figures, showing how agreement between the model and observations is related to the chosen growth threshold. Both agreement percentage and Cohen’s kappa are tracked across values for the growth threshold for both Validation Studies A and B (Figures S1 and S2).

4. Fig3: I do not quite understand why there is a distinction between the placebo and treatment group as the analysis uses metabolic modeling to explore the effects of pro-, pre- and synbiotics. I would recommend either to remove the placebo group or combine both groups. However, this is more a narrative flaw than a technical or scientific one.

Response: We agree with the reviewer that inclusion of the placebo group did not substantially contribute to the narrative of this figure. To improve clarity and focus, we have removed the placebo group from that analysis and revised the figure accordingly (Figure 3A). However, we now include results from the placebo group and the WBF-011, in terms of changes in glucose AUC and model-predicted *A. muciniphila* growth rates, to show how predicted *A. muciniphila* growth is associated with improved glucose control both within the WBF-011 treatment group and across groups (Fig. 3C-E).

5. FIg5A: I would suggest it would be more informative to provide a bar chart of percentage of samples engrafted per condition and bacterium, instead of the "heatmap".

Response: We agree. We have made the suggested change in the updated figure (Figure 5E).

6. l313-321: you discuss the shortcoming of missing strain level resolution due to lack of resolution in the databases and advice to resolve these in the future. I think it is advisable to mention here metabolic modeling reconstruction frameworks, which could be used to derive strain level metabolic models, like pathway tools, CarveMe or gapseq.

Response: The reviewer makes a great point, and we agree that it is appropriate to highlight the availability of reconstruction frameworks (e.g., Pathway Tools, CarveMe, gapseq) for building strain-level metabolic models. In the present study, however, we elected not to pursue *de novo* strain-level reconstructions for two reasons. First, the AGORA GEMs used in our MCMMs have undergone some degree of curation and validation, which would not be feasible for newly reconstructed models without substantially expanding the experimental scope of our work. Second, our modeling strategy relies on species-level GEMs to represent endogenous community members; incorporating strain-level reconstructions for the probiotic would create a mismatch in taxonomic resolution. We have added this note to the revised manuscript (lines 340-349).

7. l394: "A tradeoff parameter of 0.99 was applied..." this is a very high tradeoff parameter, when compared to results of the original paper introducing cooperative tradeoff metabolic modeling. Could you please explain, why this high trade-off parameter was used, instead of something recommended like 0.5-0.7?

Response: In general, the preference is to use as high a tradeoff parameter as possible (i.e., allowing the system to get as close as it can to optimal community growth), while retaining growth of most of the taxa in a sample. For this reason, depending on the community structure and dietary context, optimal tradeoff parameters can differ. In this case, >80% of taxa were able to grow at a fairly high tradeoff parameter of 0.99. Additionally, lower tradeoff values lead to increased leniency, resulting in a higher proportion of taxa growing. This can be helpful for modeling community-scale metabolism, but for determining growth success of individual taxa, increased leniency can result in a trivial result in which all species grow. Finally, we selected our tradeoff parameter by looking at the average growth fraction in Validation Study A, and we used the same value on Validation Study B, to avoid overfitting. We have added discussion of these points to the revision (Lines 489-492).
