# Supplementary material for: Metabolic modeling reveals determinants of prebiotic and probiotic treatment efficacy across multiple human intervention trials
Source: PLoS Biol. 2026 Feb 19;24(2):e3003638. doi: 10.1371/journal.pbio.3003638 (PMC12919772; doi:10.1371/journal.pbio.3003638)
Supplement: Attachment — Submitted filename: editor_response_2.docx [file pbio.3003638.s014.docx]

Editorial comments in **black**, response text in **blue**.

Editorial Revision Response

1) Please provide your detailed Financial Disclosure statement. This is published with the article. It must therefore be completed in full sentences and contain the exact wording you wish to be published.
- State what role the funders took in the study. If the funders had no role in your study, please state: “The funders had no role in study design, data collection and analysis, decision to publish, or preparation of the manuscript.
-If any authors received a salary from any of your funders, please state which authors and which funders.
- Please ensure that the funders and grant numbers match between the Financial Disclosure field and the Funding Information tab in your submission form. Note that the funders must be provided in the same order in both places as well. Currently, FD and FI out of order.

**Response:** I am confused by this request, but I tried my best to clean this section up.

Here is the financial disclosure:

“This study was funded, in part, by a research grant from Pendulum (to SMG), the manufacturer of the synbiotic tested in Perraudeau et al. (2020). This work was also supported by the National Institute of Diabetes and Digestive and Kidney Diseases (NIDDK) of the National Institutes of Health (NIH) under award number R01DK133468, and by a Global Grants for Gut Health Award from Nature Portfolio and Yakult (to SMG). The funders had no role in the study design, data collection and analysis, decision to publish, or preparation of the manuscript. “

Here is the Competing Interests Statement:

“SMG is a paid member of the scientific advisory board for Thorne. This work is unrelated to Thorne, and Thorne had no involvement in the study design, data collection and analysis, decision to publish, or manuscript preparation.”

And here is the updated Acknowledgements:

“Thanks to Christian Diener and members of the Gibbons Lab for useful discussions.”

Please provide exact guidance if further edits are required.

2) We have noticed that you have uploaded Supporting Information files (S1 -S5 Data), but you have not included a list of legends. Please add a full list of legends for your Supporting Information files before or after the references list.

**Response:** Legends have been added.

3) We notice that your supplementary figures are uploaded with the file type 'Figure'. Please amend the file type to 'Supporting Information'. Please ensure that each Supporting Information file has a legend listed in the manuscript before or after the references list.

**Response:** Done.

4) We notice that your supplementary [S1-S5 figures] are included in the manuscript file. Please remove them and upload them with the file type 'Supporting Information'. Please ensure that each Supporting Information file has a legend listed in the manuscript before or after the references list.

**Response:** Done.

5) In the online submission form you indicate that your data is available "Shotgun metagenomic sequencing data and metadata from Validation Study A (clinical trial NCT03893422) are not publicly posted but are available on reasonable request from the original investigators (Perredeau et al.).." According to our Data Policy, data must either be included with the submission, be placed in a public repository, or available from a third party contact point who must not be an author on the manuscript. Please revise your data statement to a non-author institutional point of contact, such as a data access or ethics committee, Please revise your data statement to a non-author institutional point of contact, such as a data access or ethics committee, Please revise your data statement to a non-author institutional point of contact, such as a data access or ethics committee, and send this to us via return email. Please also include contact information for the third party organization and
send this to us via return email. Please also include contact information for the third party organization (this contact should be a central address and not a named person), and please include the full citation of where the data can be found.

**Response:** The owners of that data set have provided the following text, which has been added to the manuscript: “Inquiries to access the data from Validation Study A can be made at john.eid@pendulum.co and will be responded to within 10 business days.”
